# Two-stage surgical resection of a massive primary posterior mediastinal dedifferentiated liposarcoma with bilateral thoracic extension: a case report

**DOI:** 10.1097/MS9.0000000000005678

**Published:** 2026-09-09

**Authors:** Chong Zhang, Chao Feng, Yong Long, Yuhao Wang, Yanhe Su, Na Han, Guiming Hu, Jin Zhang

**Affiliations:** a Department of Thoracic Surgery, The Second Affiliated Hospital of Zhengzhou University, Zhengzhou, China; b Department of Oncology, The Second Affiliated Hospital of Zhengzhou University, Zhengzhou, China; c Department of Pathology, the Second Affiliated Hospital of Zhengzhou University, Zhengzhou, China

**Keywords:** case report, dedifferentiated liposarcoma, mediastinum, two-stage surgery

## Abstract

**Introduction and importance:**

Primary mediastinal dedifferentiated liposarcoma (DDLPS) is a rare and aggressive tumor, often diagnosed late due to nonspecific symptoms. The optimal surgical strategy for tumors with bilateral thoracic extension remains unclear.

**Case presentation:**

A 59-year-old woman presented with a 1-year history of exertional dyspnea. Chest computed tomography revealed a large posterior mediastinal mass extending into both thoracic cavities with compression of adjacent structures. A two-stage surgical approach was performed, with initial right thoracotomy followed by left thoracotomy for complete resection. Histopathology confirmed DDLPS with CDK4, MDM2, and S-100 positivity and MDM2 gene amplification.

**Clinical discussion:**

Complete surgical resection is the mainstay of treatment for mediastinal DDLPS. In cases with bilateral thoracic involvement, staged surgery may reduce operative risk and improve surgical safety compared to a single-stage approach.

**Conclusion:**

Staged surgical resection is a feasible and safe strategy for massive mediastinal DDLPS involving both thoracic cavities. Careful surgical planning is essential to achieve optimal outcomes.

## Introduction

Primary mediastinal liposarcoma (PML) is a rare mesenchymal tumor originating from adipocytes, constituting less than 1% of all mediastinal tumors^[^1^]^. The main histological subtypes are well-differentiated liposarcoma (WDLPS), myxoid liposarcoma, pleomorphic liposarcoma, and dedifferentiated liposarcoma (DDLPS). DDLPS is less common and has a poorer prognosis than WDLPS^[^2,3^]^. PML typically grows insidiously, often causing no significant symptoms in its early stages; most cases are discovered when the tumor has grown large enough to compress surrounding tissues or organs^[^4^]^. Surgical intervention remains the primary treatment for PML, although postoperative recurrence is common. The pathological type and the thoroughness of surgical excision are likely key factors affecting the prognosis^[^5^]^. Given the rarity of giant posterior mediastinal DDLPS with bilateral thoracic extension and the absence of established surgical strategies for such cases, we report this case to describe the successful application of a planned two-stage surgical approach and to discuss its potential role in the management of similarly complex patients.

HIGHLIGHTS

- Primary mediastinal DDLPS is rare and often presents at an advanced stage.
- Bilateral thoracic extension makes complete resection technically challenging.
- A two-stage surgical approach can reduce operative risk.
- Staged resection enabled complete tumor removal with negative margins.
- Individualized surgical planning is essential for complex mediastinal tumors.

## Case report

In November 2021, a 59-year-old woman presented with a 1-year history of exertional dyspnea. No other symptoms or medical history were reported. Auscultation revealed diminished breath sounds in the bilateral lower lung regions. Chest computed tomography revealed a massive tumorous lesion in the posterior mediastinum, expanding toward both lower lungs. The lesions on both sides displayed uneven density, septations, and calcifications and were contiguous within the mediastinum. The lesion in the posterior mediastinal region of the left lower lung measured approximately 16.0 × 10.0 × 6.0 cm; enveloped the esophagus, aorta, and left lower pulmonary vein; showed indistinct demarcation from the pericardium; and caused local compression atelectasis of the left lower lung. The lesion in the posterior mediastinal region of the right lower lung measured approximately 11.0 × 8.0 × 5.8 cm and encircled the right lower pulmonary vein, resulting in local compression atelectasis of the right lower lung [Fig. 1(A–C)]. Preoperative evaluation demonstrated good cardiopulmonary function, and anesthetic assessment confirmed that the patient was suitable for major thoracic surgery. Following multidisciplinary discussion involving thoracic surgeons, anesthesiologists, radiologists, and pathologists, a planned two-stage surgical strategy was adopted. Although the patient was considered fit for surgery, the anticipated complexity of prolonged bilateral thoracotomy, extensive mediastinal dissection, and the potential risks of massive blood loss, respiratory compromise, and hemodynamic instability made a single-stage operation undesirable. Although the left-sided tumor was larger and more closely associated with critical mediastinal structures, the technically less complex right-sided lesion was selected for the initial procedure because it could be removed with a lower operative risk. This approach allowed definitive histopathological diagnosis while providing sufficient time to assess the patient’s postoperative recovery and fitness before proceeding to the more technically demanding second-stage resection. Following confirmation of DDLPS and an uneventful recovery, complete resection of the left-sided tumor was performed 20 days later.

**Figure 1.**
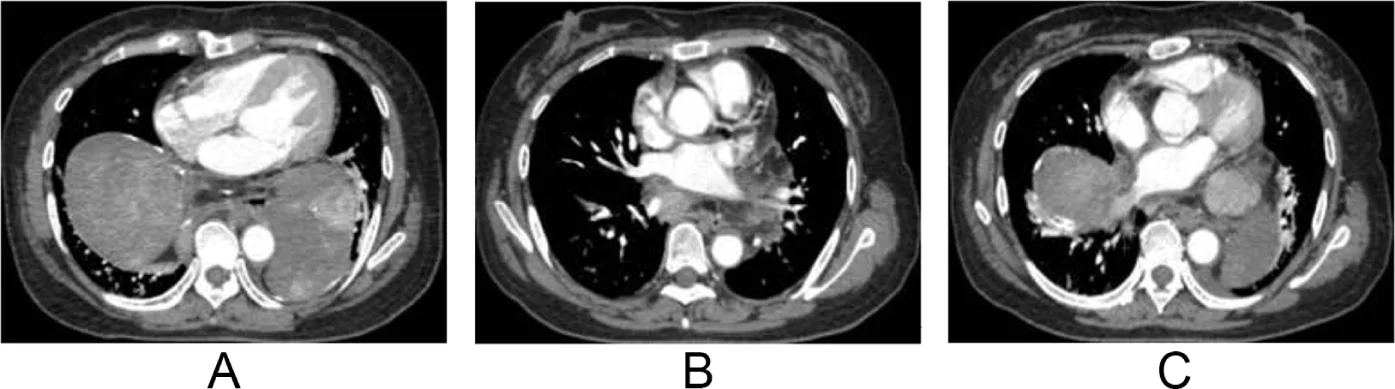
Chest computed tomography images. (A) Massive tumor in the posterior mediastinum, expansively growing toward the bilateral lower lungs. (B) Left-sided tumor enveloping the esophagus, aorta, and left lower pulmonary vein with indistinct demarcation from the pericardium. (C) Right-sided tumor encircling the right lower pulmonary vein.

The patient first underwent a right thoracotomy through the sixth intercostal space, and the operation lasted 3.5 hours. Intraoperatively, the tumor was observed in the posterior mediastinum, expanding into the right thoracic cavity and densely adherent to the right lower lung. The tumor surface was highly vascularized with numerous new blood vessels, and the tumor was firm in consistency without obvious invasion. After ligating the tumor’s feeding vessels, the right-sided portion of the tumor was excised. The gross specimen of the tumor appeared grayish-red, measured approximately 11.0 × 8.0 × 5.8 cm, exhibited a smooth surface and intact capsule, and had a firm, grayish-white cut surface with a woven appearance [Fig. 2(A)]. Microscopic examination revealed a spindle cell tumor with localized presence of lipoblasts and well-differentiated adipocytes, with parts of the tumor showing smooth muscle differentiation. The tumor cells were densely packed and moderately atypical. Immunohistochemistry results were positive for cyclin-dependent kinase 4 (CDK4), mouse double minute 2 (MDM2), and S-100 protein. Molecular pathology examination revealed MDM2 gene amplification by fluorescence in situ hybridization (FISH) [Fig. 3(A–F)]. Based on these findings, the diagnosis was considered to be DDLPS. The patient recovered uneventfully postoperatively without significant complications, and the chest drainage tube was removed on the third postoperative day. Twenty days postoperatively, after comprehensive evaluation, the patient underwent a left thoracotomy through the sixth intercostal space, and the surgery lasted 4 hours. Intraoperatively, the tumor was observed in the posterior mediastinum, expanding into the left thoracic cavity. It was characterized by abundant neovascularization, a firm texture, and dense adhesions to the left lower lung and pericardium, which precluded complete dissection. Partial pericardiectomy was performed to ensure complete tumor excision. The gross tumor specimen was grayish-red; measured approximately 16.0 × 10.0 × 6.0 cm; and exhibited a smooth surface, intact capsule, localized calcifications, and a firm, grayish-white cut surface with focal gray-red areas (Fig. 2(B)). Microscopic findings, immunohistochemistry results, and molecular pathology results were consistent with those observed in the first surgical specimen. Potentially involved lymph nodes, including the subaortic and subcarinal lymph nodes, were also cleared during the surgery, and postoperative pathologic examination revealed no lymph node metastasis. Because no related adipose tumor lesions were identified in other parts of the patient’s body, and because the tumor predominantly occupied the posterior mediastinum and exhibited a smooth surface and intact capsule, the definitive diagnosis was primary mediastinal DDLPS.

**Figure 2.**
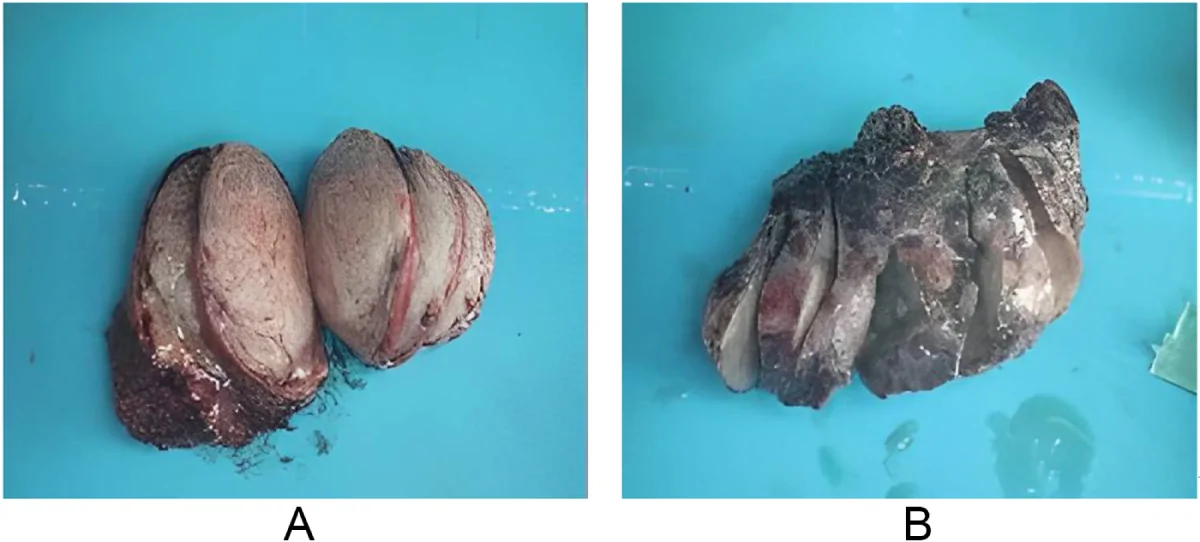
Gross appearance of the excised tumors: (A) right-sided tumor portion removed during the first surgery and (B) left-sided tumor portion removed during the second surgery.

**Figure 3.**
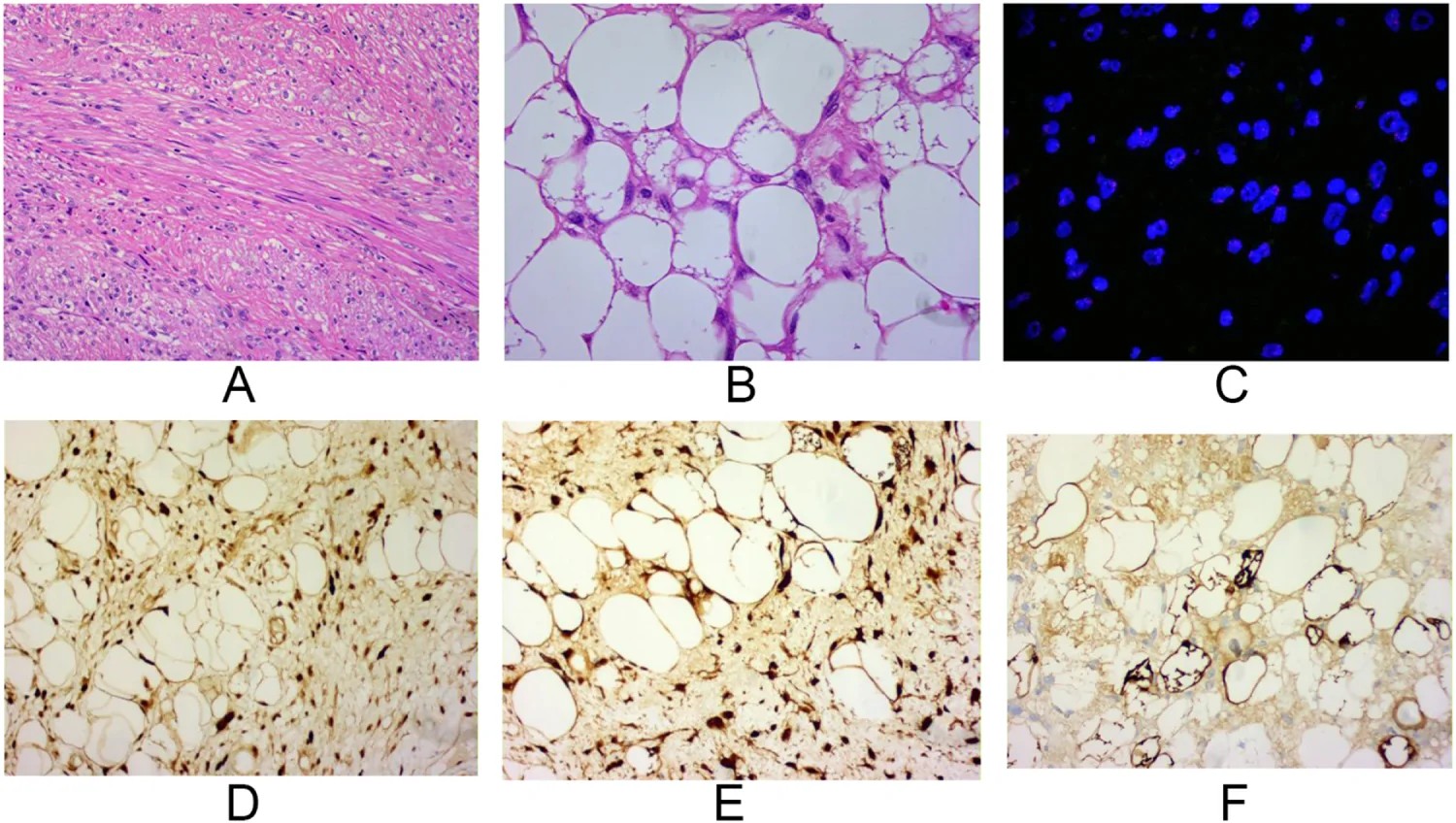
Histological features of the tumor. (A) A spindle cell component was observed under the microscope (H&E, 200×). (B) Abundant lipoblasts and well-differentiated adipocytes were seen under the microscope (H&E, 400×). (C) FISH analysis detected MDM2 gene amplification, and immunohistochemical analysis revealed positive nuclear immunoreactivity for (D) MDM2, (E) CDK4, and (F) S-100 in tumor cells. H&E, hematoxylin–eosin.

The patient had an uneventful postoperative recovery with no apparent complications. The chest drainage tube was removed on the third postoperative day, and the patient was discharged on the ninth postoperative day. After discharge, the patient underwent regular outpatient follow-up, including clinical evaluations and chest computed tomography, with no evidence of postoperative complications during follow-up. Follow-up chest computed tomography examinations at 3 and 9 months after discharge revealed no signs of tumor recurrence [Fig. 4(A and B)]. Because of financial constraints, the patient declined postoperative radiotherapy. At 12 months after surgery, the patient developed severe COVID-19 pneumonia complicated by multiple organ failure and died despite intensive supportive treatment. Importantly, there was no clinical or radiological evidence of tumor recurrence before death.

**Figure 4.**
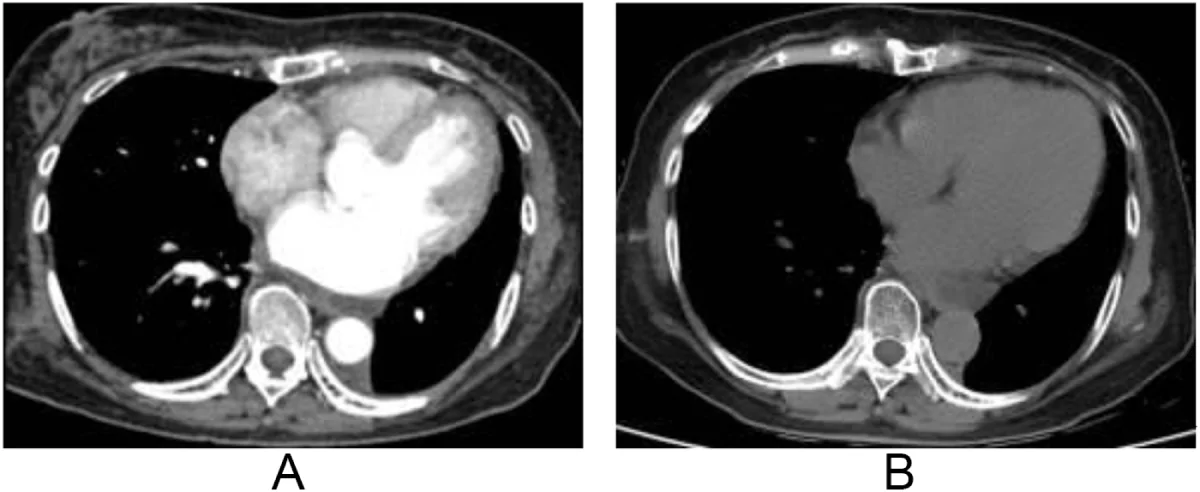
No signs of recurrence were observed in two consecutive chest CT scans: (A) three months post-surgery and (B) nine months post-surgery.

This case report complies with the CARE guidelines^[^6^]^. This study was approved by the Institutional Review Board of the Second Affiliated Hospital of Zhengzhou University (No. 2023281) and was published with written informed consent obtained from the patient and her family.

## Discussion

PMLs are predominantly found in the posterior mediastinum, accounting for 48% of cases, followed by the anterior mediastinum (28%) and the middle mediastinum (11%)^[^7^]^. Typically, PMLs exhibit no significant symptoms in their early stages, making early detection challenging until they grow large enough to compress surrounding tissues and organs.

WDLPS and DDLPS share significant cytogenetic similarities. High-level amplification of genes in the chromosome 12q13-15 region is a common molecular disorder in both diseases, resulting in overexpression of CDK4 and MDM2. Overexpression of MDM2 inhibits p53-induced transcription and leads to ubiquitination and degradation of p53, processes that contribute to tumor development^[^8^]^. Furthermore, DDLPS exhibits additional genetic abnormalities, with more frequent and higher-level amplifications in the 12q13-15 chromosomal region, as well as co-amplifications at 6q23 and 1p32, indicating a higher degree of genetic heterogeneity^[^9^]^. These molecular alterations are not only important diagnostic hallmarks of DDLPS but also correlate with its biological behavior. Compared with WDLPS, DDLPS exhibits a substantially higher propensity for local recurrence even after complete surgical resection, whereas distant metastasis remains relatively uncommon. Therefore, complete surgical excision followed by long-term postoperative surveillance remains the cornerstone of management. In addition, S-100 positivity supports adipocytic differentiation and complements MDM2 and CDK4 in establishing the pathological diagnosis of DDLPS^[^9,10^]^.

These factors likely contribute to the more aggressive nature, higher recurrence rate, and poorer prognosis of DDLPS compared with WDLPS. Certain cases of DDLPS originate from WDLPS, with the transformed state potentially indicating a more unfavorable prognosis. Suto *et al*^[^10^]^ reported that, because of diagnostic delays, retroperitoneal liposarcomas adjacent to the testicles may be diagnosed as DDLPS, precluding curative surgery and ultimately resulting in a poorer prognosis. Studies have revealed a dedifferentiation rate of 6% in WDLPS of the extremities, whereas retroperitoneal and inguinal cases exhibit rates of 17% and 29%, respectively^[^11^]^. However, because of the scarcity of cases originating in the mediastinum, few large-scale studies on the transformation of thoracic or mediastinal WDLPS into DDLPS have been performed to date. Typically, WDLPS lacks lipoblasts and does not exhibit myogenic differentiation, whereas DDLPS often displays features of undifferentiated pleomorphic sarcoma or spindle cell sarcoma, with lipoblasts and myofibroblasts commonly present. Recent studies have confirmed that DDLPS with myogenic components, particularly those with rhabdomyoblastic differentiation, have a poorer prognosis^[^9,12^]^.

In recent years, the use of doxorubicin monotherapy or combination regimens with ifosfamide for treating DDLPS has shown an efficacy of only 10–23%, and such treatment does not significantly improve survival^[^13,14^]^. Certain studies have posited that postoperative radiotherapy decreases the risk of local recurrence of liposarcomas (encompassing retroperitoneal and non-retroperitoneal tumors) while yielding disparate effects on overall survival^[^15^]^. Given that MDM2 and CDK4 are key factors in the pathogenesis of WDLPS and DDLPS, drugs targeting MDM2 and CDK4 have been introduced. However, MDM2 inhibitors have been associated with an adverse event rate of up to 40% in some trials^[^16^]^. A phase Ib study by Abdul Razak *et al*^19^ reported that CDK4/6 and MDM2 inhibitors, when used as single agents, demonstrated promising early clinical results. The combination of siremadlin (an MDM2 inhibitor) and ribociclib (a CDK4/6 inhibitor) exhibited synergistic effects, showing early antitumor activity and a manageable safety^[^17^]^. Although surgery remains the cornerstone of treatment for localized disease, advances in molecular-targeted therapy may provide additional treatment options for patients with unresectable, recurrent, or metastatic DDLPS in the future.

To better place the present case in the context of the existing literature, previously reported surgically treated mediastinal DDLPSs are summarized in Table 1. Among the three posterior mediastinal DDLPSs reported by Ortega *et al*, all patients underwent single-stage resection. The only patient who achieved R0 resection had the smallest tumor and remained alive without disease at 24 months, whereas the remaining patients had persistent disease or were lost to follow-up^[^18^]^. Likewise, Miura *et al* reported four surgically treated mediastinal DDLPSs, all managed by single-stage resection, three of whom developed local recurrence during follow-up despite initial R0 resection^[^19^]^. Collectively, these reports highlight that although complete surgical excision remains the cornerstone of treatment, local recurrence is frequent even after R0 resection, underscoring the need for meticulous long-term surveillance^[^18–20^]^.

**Table 1:** Comparison of previously reported mediastinal dedifferentiated liposarcoma (DDLPS) cases and the present case.

Study	Case	Age/sex	Tumor size (cm)	Bilateral thoracic extension	Surgical strategy	R0 resection	Outcome
Ortega *et al* (2015)	13	75/M	14 × 10 × 8	No	Single-stage	No	AWD, 6 months
	14	66/M	9 × 8 × 5	No	Single-stage	Yes	A&W, 24 months
	15	53/F	23 × 20 × 12	No	Single-stage	NR	Lost to follow-up
Miura *et al* (2018)	1	45/F	12.1 × 8.2 × 5.8	No	Single-stage	Yes	Local recurrence (51 months), repeat resection
	2	62/F	8.8 × 6.6	No	Single-stage	Yes	Local recurrence (28 months), proton therapy
	3	84/M	6.6 × 3.0	No	Single-stage	Yes	Local recurrence (28 months), repeat resection
	4	75/M	20	Yes	Single-stage (bilateral thoracotomy)	Yes	No recurrence (3 months)
Present case	1	59/F	16 × 10 × 6 + 11 × 8 × 5.8	Yes	Two-stage surgery	Yes	No recurrence (9 months); died of COVID-19 pneumonia at 12 months

AWD, alive with disease; A&W, alive without evidence of disease; NR, not reported.

When taken together with previous reports (Table 1), although single-stage resection remains the standard surgical strategy for most mediastinal DDLPSs, a staged approach may represent a reasonable alternative for exceptionally large tumors with bilateral thoracic extension when prolonged surgery is expected to carry substantial perioperative risk^[^21,22^]^. In the present case, the exceptionally large tumor with bilateral thoracic extension and its close relationship with multiple vital mediastinal structures made single-stage resection technically challenging. Therefore, a planned two-stage approach was adopted to facilitate safe R0 resection while reducing operative burden. Although complete tumor removal was achieved, the patient subsequently died of COVID-19-related pneumonia with multiple organ failure 12 months after surgery, precluding assessment of long-term oncological outcomes.

In conclusion, this case demonstrates that staged resection may be a feasible surgical option for carefully selected patients with exceptionally large posterior mediastinal DDLPS extending into both thoracic cavities, when complete single-stage resection is considered technically demanding or associated with excessive perioperative risk. To our knowledge, this is the first reported mediastinal DDLPS successfully managed using a planned two-stage surgical strategy. Although the comparative effectiveness of staged versus single-stage resection remains uncertain, this case expands the current surgical experience and may provide a useful reference for future management of similarly complex patients.

## Data Availability

The data that support the findings of this study are available from the corresponding author upon reasonable request.
